# Neo-antigen tumor vaccination depends on CD4-licensing conveyed by adeno-associated virus like particles

**DOI:** 10.1016/j.ymthe.2025.07.014

**Published:** 2025-07-16

**Authors:** Lasse Neukirch, Silke Uhrig-Schmidt, Katharina von Werthern, Alexandra Tuch, Joscha A. Kraske, Yanhong Lyu, Benedicte Lenoir, Stefan B. Eichmüller, Marten Meyer, Inka Zörnig, Dirk Jäger, Patrick Schmidt

**Affiliations:** 1 Clinical Cooperation Unit “Applied Tumor Immunity”, German Cancer Research Center (DKFZ), Im Neuenheimer Feld 460, Heidelberg, Germany; 2 Department of Medical Oncology, National Center for Tumor Diseases (NCT), University Hospital Heidelberg, Im Neuenheimer Feld 460, Heidelberg, Germany; 3 GMP and T Cell Therapy Group, German Cancer Research Center (DKFZ), Im Neuenheimer Feld 280, Heidelberg, Germany

**Keywords:** AAVLP, neo-antigen, vaccine, immunotherapy, CD4 licensing

## Abstract

Personalized treatment has become a realistic option for tumor patients, accelerated by significantly reduced sequencing costs of tumor genomes and advances in vaccine formulations. The druggability of cancer neo-antigens caused by individual mutations is centered in this effort. We here use an adeno-associated virus (AAV)-based virus-like particle (VLP) platform to compose a neo-antigen-specific protein vaccine that is effective in a murine prevention and treatment setting. Furthermore, we show that CD4^+^ T cell responses that are provided by the AAV capsid are crucial for effective murine melanoma treatment. To uncover the optimal composition of a peptide vaccine we de-linked major histocompatibility complex (MHC) class II helper peptides from the capsid and formulated an efficient neo-antigen-specific vaccine, which showed the independence of CD4^+^ T cell response from tumor sequences. The findings are supported by clinical data of neo-antigen-vaccinated tumor patients. Our results punctuate on the significance of MHC class II epitopes for CD8^+^ T cell responses and suggest a future use of AAVLPs as neo-epitope vaccines in personalized cancer treatments.

## Graphical abstract

**Figure:**
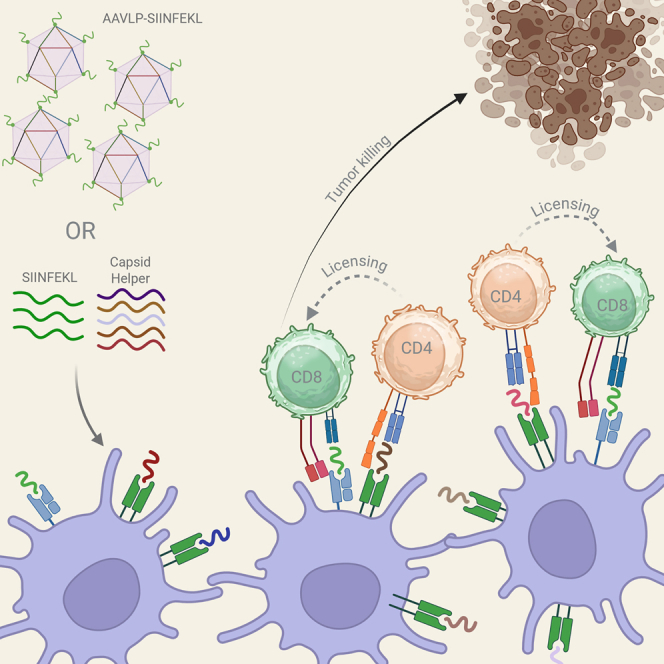

## Teaser

By using an aggressive mouse melanoma model in combination with clinical data, Schmidt and colleagues show the sequence independency of CD4 licensing during vaccination.

## Introduction

Cancer vaccination addressing neo-antigens based on the mutanome of an established tumor is the most personalized immunotherapeutic approach so far.^1^ First clinical results in melanoma^2^^,^^3^ and pancreatic cancer^4^ indicate the induction of a long-lasting and *de novo*-generated response caused by an individually formulated mRNA vaccine. Composition of these vaccines includes predicted human leukocyte antigen (HLA) class II and class I epitopes to induce a robust mixed CD4^+^ and CD8^+^ T cell response, which is reported to be a crucial factor for success.^5^ Several studies in mice have shown that CD4^+^ T cells are required for secondary responses of CD8^+^ T cells,^6^^,^^7^ as well as the initial priming of the cytotoxic cells via conventional type 1 dendritic cells.^8^ These findings support the necessity to formulate an anti-cancer vaccine with epitopes from both classes. However, these studies only focused on on-target sequences of HLA class II epitopes and did not investigate the role of non-tumor epitope sequences.

Virus-like particles (VLPs) have a long standing history in vaccine research.^9^ They are multi-protein structures that resemble intact viruses but do not contain a viral genome.^10^^,^^11^ While VLPs are intentionally used to induce strong antibody responses and, therefore, are clinically established in cancer prevention,^12^ viral infection,^13^ and chronic or acute inflammation,^14^ the biology and size of the particles allow an uptake in antigen-presenting cells (APCs) of the lymphatic system.^15^ The adeno-associated virus (AAV) is a non-enveloped, single-stranded DNA virus with a relatively small size of 25 nm.^16^^,^^17^ Belonging to the genus of *Dependovirus* in the family of *Parvoviridae*, AAV replication depends on the presence of helper viruses, such as adenovirus or herpesvirus, which provide genes for genome replication and viral assembly. Owing to its non-pathogenicity in humans and ability to transduce a broad spectrum of cells, AAV became an established vector for gene therapy,^16^^,^^18^ leading to the first approval by the US Food and Drug Administration in 2017.^17^ Despite their frequent use as gene therapy vectors, several studies tested AAVs for vaccination purposes. Most of these approaches were based on genetic immunotherapies, in which a target antigen is encoded by the vector. Upon transduction of cells, antigens are expressed and presented on major histocompatibility complex (MHC) to evoke cellular responses by T cells or are released into circulation to induce antibody secretion by B cells. This strategy was tested in several preclinical mouse studies targeting model antigens like ovalbumin (OVA)^19^ or antigens from viruses,^20^ parasites,^21^ and cancer cells.^22^ In addition, a vaccine candidate against simian immunodeficiency virus was tested in macaques,^23^ and two HIV vaccines reached clinical trials.^24^^,^^25^ In both preclinical and clinical studies, explicit antibody as well as cellular immune responses were observed. Comparable with a vaccination approach, the generation of T cell responses to the CD8^+^ T cell epitope SIINFEKL was tested upon presentation on AAVs.^26^ These studies were primarily initiated as a model system because human gene therapy trials showed vector cytotoxic T cell responses that were previously not observed in mice.^27^ To increase immunogenicity in murine model systems and test capsid antigen presentation *in vitro* and *in vivo*, the additional SIINFEKL antigen was displayed on the AAV surface.^28^ However, these studies followed the final aim of preventing immune responses, rather than capitalizing them for vaccination approaches.

In this study we aimed to investigate whether the anti-tumor-specific CD8^+^ T cell responses can be unlinked from anti-tumor CD4^+^ specificity. By constructing AAVLPs that display HLA class I shared and neo-antigens, we show their functionality in an established murine tumor model that is reliant on viral HLA class II epitopes. This finding is furthermore supported by our own retrospective clinical data on personalized cancer vaccination.

## Results

### SIINFEKL-displaying AAVLPs induce CD8^+^ T cell responses and prevent tumor development

**Figure 1:**
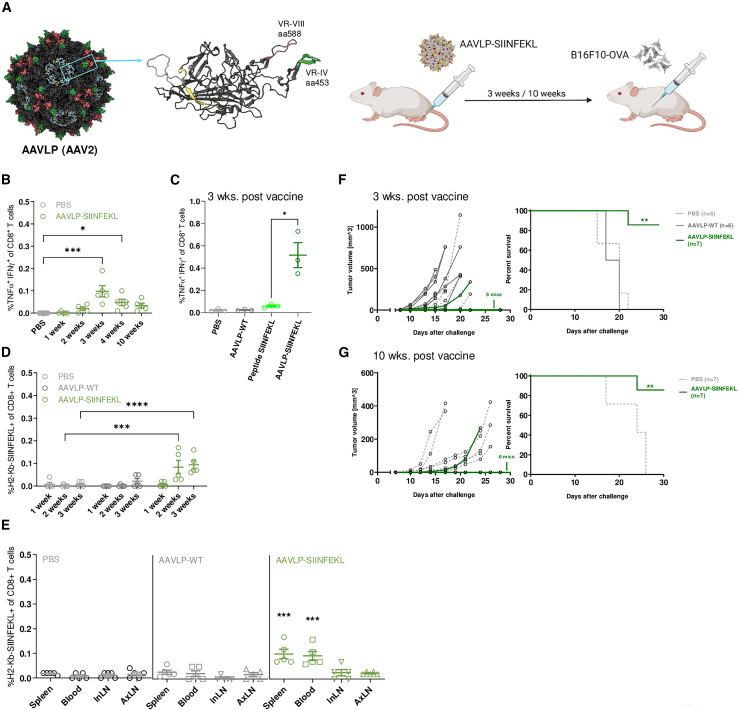
AAVLP-SIINFEKL vaccines prevent tumor formation by CD8^+^-mediated rejection (A) For generation of antigen-displaying AAVLPs, target sequences were inserted into the *cap* gene. In case of serotype 2 AAVLPs (AAV2), insertion sites were in the VR-VIII loop (around aa588) or the VR-IV loop (around aa453) of the VP1 protein. All mice were injected with 5.0E+11 AAVLPs in Montanide ISA 51, s.c. into the hock, followed by s.c. injection of 2.0E+5 melanoma cells into the left flank 3 or 10 weeks later. (B) Analysis of CD8^+^ T cell responses at different time points after vaccination with AAVLP-SIINFEKL. The level of antigen-specific T cells was determined by IFN-γ and TNF-α staining after stimulating splenocytes with SIINFEKL peptide, followed by FACS (*n* = 5). (C) CD8^+^ T cell response analysis of AAVLP-vaccinated mice in comparison with peptide-vaccinated mice (*n* = 3). SIINFEKL peptide was administered via the same route as AAVLP in equimolar amounts (0.1 μg). (D) Antigen-specific CD8^+^ T cells in the blood of AAVLP-SIINFEKL-vaccinated mice were determined weekly by FACS after staining with fluorescent-labeled H2-Kb-SIINFEKL tetramer. Mice injected with PBS or AAVLP-WT served as controls (*n* = 5). (E) Antigen-specific CD8^+^ T cells in AAVLP-SIINFEKL-vaccinated mice were analyzed after 3 weeks by tetramer staining in spleen, blood, inguinal lymph nodes (InLN), and axillary lymph nodes (AxLN). Mice injected with PBS or AAVLP-WT served as controls (*n* = 5). (F and G) Individual tumor growth curves in each mouse of the AAVLP-WT and AAVLP-SIINFEKL vaccinated group after challenge with B16F10-OVA melanoma cells. For comparison, the growth curves of PBS-injected mice are shown as dashed, light gray lines. Survival of AAVLP-SIINFEKL-vaccinated mice compared with the control groups (*n* = 6 for PBS, *n* = 7 for AAVLP groups). Statistical analysis on survival was performed using the log rank (Mantel-Cox) test. ∗∗*p* < 0.01. Horizontal bars indicate the mean of each group with SEM. Significant differences to negative controls were determined using an one-way ANOVA (B and C) or two-way ANOVA (E and G) with a Dunnett’s multiple comparisons test. Asterisks indicate significant difference to the PBS group with ∗*p* ≤ 0.05, ∗∗∗*p* ≤ 0.001, and ∗∗∗∗*p* ≤ 0.0001.

We have engineered capsid-modified AAV-2 particles by genomic insertion of the DNA sequence encoding the SIINFEKL peptide in either the VR-VIII or the VR-IV loop of the VP1 protein (Figures 1A and S1A). A vaccination scheme was established in immunocompetent C57BL/6 mice by comparison of different injection routes, doses, and adjuvants. Vaccine responses were determined by the frequency of CD8^+^ T cells secreting interferon (IFN)-γ and tumor necrosis factor (TNF)-α derived from splenocytes after vaccination and after co-incubation with peptide pulsed autologous APCs. Subcutaneous hock injection induced T cell responses more frequently compared with tailbase and intramuscular injections and the use of Montanide ISA51 as an adjuvant was more effective than c-di-AMP, CpG, and combinations thereof (Figures S1B and S1C). We defined 5.0E+11 VLPs to be an effective dose (Figure S2), which induced a significant response to the AAVLP-SIINFEKL vaccine which peaked at 3 weeks after vaccination, after which it gradually decreased (Figure 1B). At 3 weeks the AAVLP vaccine outperforms short peptide vaccination using SIINFEKL alone (Figure 1C). H2-Kb-SIINFEKL tetramer staining showed that specific CD8^+^ T cells are already generated in blood at 2 weeks after vaccination, although T cells from spleens are not responsive to pulsed APCs at that time (Figure 1D). Analysis of lymphoid organs revealed that tetramer-specific T cells are generated in the spleen and blood of vaccinated mice but not in the inguinal or axillary lymph nodes (Figure 1E). To test the functional consequences of the AAVLP vaccine, we challenged mice by subcutaneous (s.c.) injection of B16F10 melanoma cells stably expressing the OVA protein at 3 weeks and 10 weeks after vaccination. In both cases, we observed a preventive effect of the AAVLP-SIINFEKL vaccine in six of seven animals (85%), which led to abrogated tumor formation and consequently longer survival compared with unvaccinated or non-engineered AAV2-wild-type (WT)-vaccinated mice (Figures 1F and 1G). To test the broad applicability and means to boost potency, we also made use of the AAVLP vaccination strategy for the viral epitope LCMV (Figure S3A) and showed feasibility of double insertions at loop IV and VII in parallel with SIINFEKL and the T helper type 1-stimulating peptide J-ICBL^29^ (Figure S3B).

### Neoantigen-AAVLPs delay tumor formation

**Figure 2:**
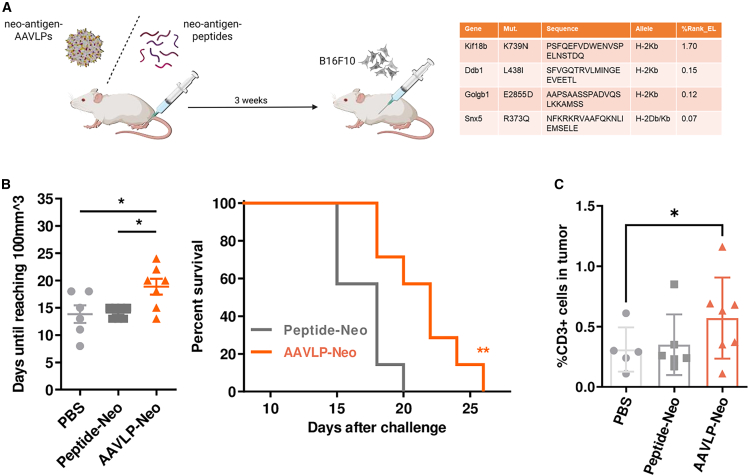
Reduced tumor growth after neo-antigen-AAVLP vaccination (A) Vaccination scheme and table of neo-epitopes chosen for vaccination. The corresponding mutation is displayed in the center of the respective 21mer peptide. (B) Mice were vaccinated simultaneously with four different neoantigen-displaying AAVLPs (AAVLP-Neo) at a dose of 5.0E+11 capsids each. AAVLPs displayed predicted MHC class I epitopes in the VR-IV loop and were injected s.c. into the hock. Mice injected with PBS or 21-aa-long peptides of the same epitopes (Peptide-Neo) served as controls. Tumor growth was analyzed every 2–3 days and mice were euthanized upon reaching a humane endpoint. The graph shows the number of days between tumor challenge and reaching a volume of 100 mm^3^ for each animal. Horizontal bars indicate the mean of each group (*n* = 7) with SEM. Survival curves showing the percentage of live animals within a group (*n* = 7) on each day. (C) Infiltration of T cells into tumor tissue. Tumors were isolated and brought into single cell suspension. The percentage of CD3^+^ T cells were analyzed by flow cytometry. Horizontal bars indicate the mean of each group (*n* = 5 for PBS, *n* = 6 for peptide-neo; *n* = 7 for AAVLP-neo) with SEM. Significant differences between groups were determined using an one-way ANOVA with a Tukey’s multiple comparisons test or a Mantel-Cox test (Kaplan-Meier-Plot). Asterisks indicate significant difference with ∗*p* ≤ 0.05, ∗∗*p* ≤ 0.01, and ∗∗∗*p* ≤ 0.001.

In this study, we use B16F10 melanoma cells as a model cell line due to its aggressiveness and the frequent occurrence of mutations that can be exploited as neo-antigens.^30^ We have chosen the peptide sequences of *Kif18b*(K739N), *Ddb1*(L438I), *Golgb1*(E2855D), and *Snx5*(R373Q) to serve as vaccine candidates based on their predicted NetMHCPan 4.1 eluted ligand rank position in the H2-Kb allele (Figure 2A) and their ability to form intact particles when inserted into the AAV2 framework (data not shown). We have used the prophylactic vaccination scheme as described before with the difference of using the parental B16F10 cell line. We have compared a composite of all four neo-antigen AAVLPs with an equimolar mixture of the respective 21mer peptides followed by tumor cell injection 3 weeks after vaccination. Although the induction of CD8^+^ responses in the spleen was significantly altered for only one AAVLP-neoantigen (Figure S4), in comparison with PBS-injected mice we observed a significant delay of tumor growth in the AAVLP-Neo group but not in the peptide vaccination group (Figures 2B and S5A) that consequently led to a significant longer survival of AAVLP-vaccinated mice. At the point of euthanization, the grown tumors were explanted and the cellular composition was analyzed by fluorescence-activated cell sorting (FACS). We detected a significant enrichment of CD3^+^ T cells in the AAVLP treatment group when compared with the sham injection group but no change when the peptide-neo-antigen vaccine was administered. Further analysis revealed a trend toward higher CD4^+^ and CD8^+^ T cell infiltration although being not statistically significant (Figure S5B). Notably, the number of infiltrated CD3^+^ T cells inversely correlated with the size of outgrown tumors after vaccination (Figure S5C).

### CD4^+^ T cell epitopes in AAVLP vaccines are required for antigen-specific CD8^+^ T cell responses

**Figure 3:**
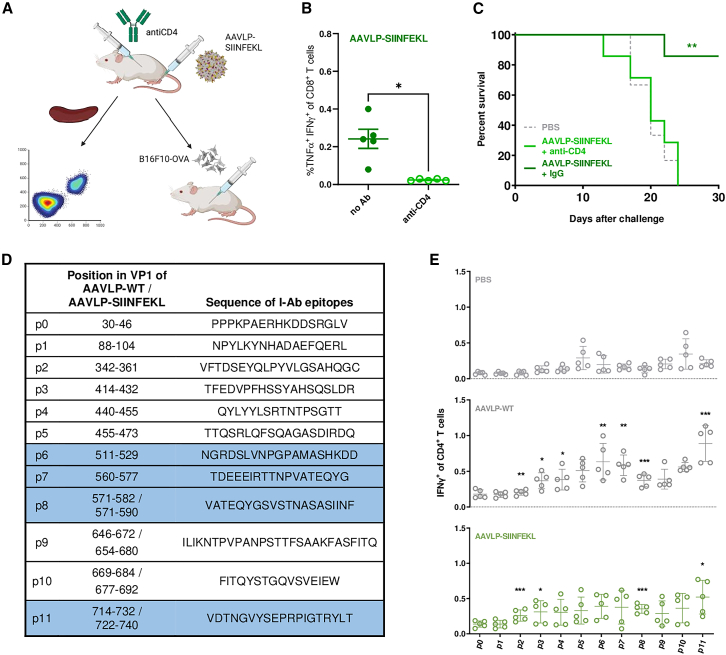
Role of CD4^+^ T cells in induction of antigen-specific CD8^+^ T cell responses and identification of AAVLP helper epitopes (A) Experimental design. (B) By injecting the anti-CD4 antibody GK1.5, CD4^+^ T cells were depleted during s.c. hock vaccination with AAVLP-SIINFEKL. Vaccinated mice without CD4^+^ T cell depletion served as controls. Antigen-specific CD8^+^ T cell responses were determined after 3 weeks by stimulating splenocytes with SIINFEKL peptide, followed by intracellular staining of activation markers TNF-α and IFN-γ. Horizontal bars indicate the mean of each group (*n* = 5) with SEM. Significant differences were determined using a two-tailed t test. (C) Mice were injected with a depleting anti-CD4 antibody or an immunoglobulin G (IgG) isotype control during vaccination with AAVLP-SIINFEKL. PBS-injected mice served as negative controls. Three weeks after vaccination, mice were challenged by injection of 2.0E+05 B16F10-OVA cells s.c. into the flank. Tumor growth was analyzed every 2–3 days and mice were euthanized upon reaching an ethical endpoint. The graph shows the percentage of live animals within a group (*n* = 6 for PBS, *n* = 7 for AAVLP groups) on each day. (D) Potential AAVLP helper epitopes in AAVLP-SIINFEKL were predicted using NetMHCII 2.3 and NetMHCIIpan 4.0. The table shows the peptide sequences with the corresponding position in AAVLP-WT or AAVLP-SIINFEKL. Helper epitopes used in further vaccination studies are marked in blue. (E) Mice were injected with 5.0E+11 capsids of AAVLP-WT or AAVLP-SIINFEKL s.c. into the hock. PBS-injected mice served as controls. Three weeks after injection, AAVLP-specific CD4^+^ T cell responses were analyzed by stimulating splenocytes with predicted MHC class II epitope peptides (p1–p11). Peptide p0, with no predicted MHC class II binding, was included as a negative control. Activated CD4^+^ T cells were analyzed by intracellular staining of activation marker IFN-γ. Horizontal bars indicate the mean of each group (*n* = 5) with SEM. Significant differences to the negative control peptide p0 were determined using a one-way ANOVA with a Dunnett’s multiple comparisons test. Asterisks indicate significant difference with ∗*p* ≤ 0.05, ∗∗*p* ≤ 0.01, and ∗∗∗ (*p* ≤ 0.001.

To investigate the influence of CD4^+^ T cell responses on the outcome of AAVLP vaccination, we treated mice with a CD4-depleting antibody before administering the vaccine (Figure 3A). After CD4^+^ T cell depletion, the AAVLP vaccination scheme did not induce detectable CD8^+^ T cell responses in the spleen upon SIINFEKL stimulation as observed before (Figure 3B). Depletion of B cells in turn did not impede the induction of CD8^+^ T cell responses (Figure S6). In a tumor challenge experiment, the CD4^+^ T cell depletion setting also abrogated the AAVLP vaccination outcome as CD4^+^ T cell-depleted mice showed the same survival rate as untreated littermates while antibody isotype pre-treated mice responded to the AAVLP immunization as seen before (Figure 3C). All animals in this experiment remained in a healthy condition while being CD4^+^ depleted until tumor challenge. Signs of autoimmune disease were not observed, as it may be imputed by a possible CD4^+^ regulatory T cell depletion in this setting. We next analyzed the AAVLP capsid proteome *in silico* for the occurrence of potential MHC class II (I-Ab) epitopes that may serve as CD4^+^ T cell receptor-specific targets. We identified 11 peptides of variable length as the top EL rank candidates of the NetMHCIIpan 4.0 analysis and named them p1–p11 (Figure 3D). A potentially non-binding peptide of the capsid sequence was labeled as negative control for further experiments (p0). Interestingly, 10 of the 11 top candidates contain no sequence bricks from the SIINFEKL protein and, in the case of p8, 4 amino acids (aa) belong to the epitope sequence but are not found in the core of the predicted binding peptide. We then assessed the functional responses of AAVLP vaccination-induced CD4^+^ T cells to the predicted capsid epitopes. After AAVLP-WT or AAVLP-SIINFEKL administration, IFN-γ-secreting cells were detected for seven peptides and four peptides, respectively (Figure 3E).

### Implementation of MHC II epitopes improves therapeutic vaccination

**Figure 4:**
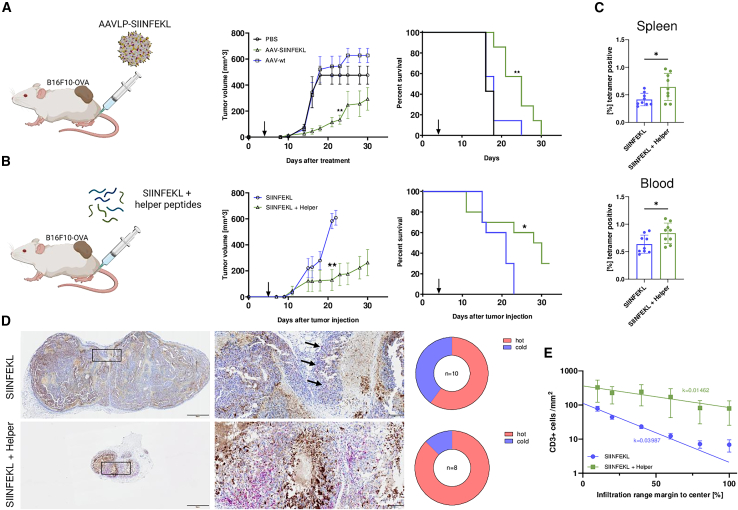
Therapeutic effects of AAVLP vaccination and a substituted peptide vaccine (A) Mice were injected with 1.0E+5 B16F10-OVA melanoma cells s.c. into the flank at day 0. At day 4 when tumors were palpable, all mice received one dose of 5.0E+11 capsids AAVLP-SIINFEKL s.c. into the hock; mice receiving PBS or AAV-WT capsids served as control (*n* = 7). Tumor growth was monitored ever 2–3 days and mean tumor growth with SEM is plotted. Survival curves showing the percentage of live animals within a group (*n* = 7) on each day. (B) Tumor-bearing mice (*n* = 10) as in (A) were treated with one dose of SIINFEKL peptide alone (0.1 μg) or SIINFEKL peptide + AAV capsid helper peptides (p6, p7, p8, and p11; 0.1 μg each) s.c. into the hock at day 4 after tumor injection. Mean tumor growth and survival are plotted as before. (C) Antigen-specific CD8^+^ T cells in the spleen of peptide-treated mice were determined at day of euthanization or day 32 by FACS staining with fluorescent-labeled H2-Kb-SIINFEKL tetramer. (D) Immunohistochemical stains of outgrown tumors in (C) were performed using the anti-CD3 mAb SP7 and BOND Polymer Refine Red Detection kit. Full slides were scanned and analyzed using the HALO software. Scale bars, 1 mm (left) or 50 μm (right). Arrows depict stained CD3^+^ cells. Determination of immunological hot tumors was set by the number of CD3^+^ cells per square millimeter. (E) Infiltration rate of tumors in peptide treated mice was categorized in percentages of distance to the tumor center and number of CD3^+^ cells/mm^2^ in each category. Exponential regression analysis (One Phase Decay) was performed and the growth rate k plotted. Horizontal bars indicate the mean of each group with SEM. Significant differences between groups were determined using an one-way ANOVA with a Tukey’s multiple comparisons test (all grouped plots), an unpaired two-tailed t test (endpoint plots) a Mantel-Cox test (Kaplan-Meier plot) or a paired two-tailed t test (infiltration plot). Asterisks indicate significant difference with ∗*p* ≤ 0.05; ∗∗*p* ≤ 0.01, and ∗∗∗*p* ≤ 0.001.

**Figure 5:**
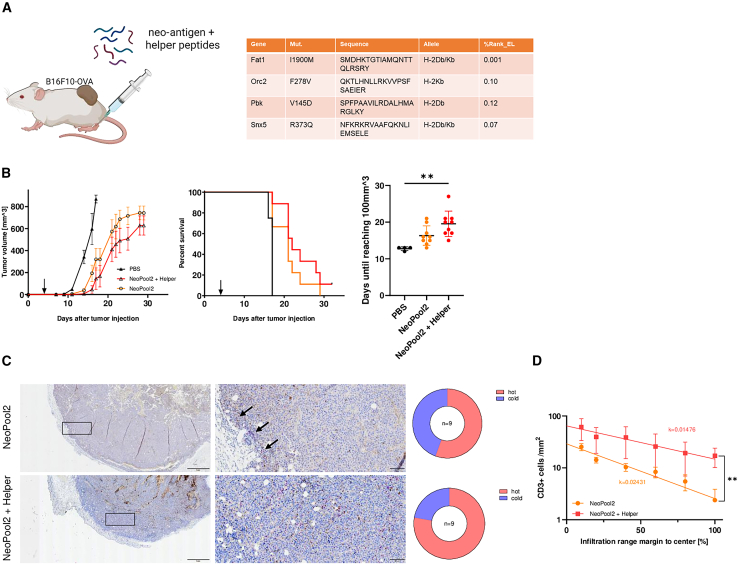
Reduced tumor growth after therapeutic helper epitope-substituted neo-antigen vaccination (A) Mice were injected with 1.0E+5 B16F10-OVA melanoma cells s.c. into the flank at day 0. At day 4 when tumors were palpable, all mice received one dose of a pool of neo-antigen peptides (100 μg each) as depicted in the table or the same pool substituted with helper peptides (p6, p7, p8, and p11; 0.1 μg each) s.c. into the hock; mice receiving PBS served as control (*n* = 10). (B) Tumor growth was monitored every 2–3 days and the mean tumor growth with SEM is plotted. Survival curves showing the percentage of live animals within a group on each day. Right graph shows the number of days between tumor challenge and reaching a volume of 100 mm^3^ for each animal. (C) Immunohistochemical stains of outgrown tumors in C were performed using the anti-CD3 mAb SP7 and BOND Polymer Refine Red Detection kit. Full slides were scanned and analyzed using the HALO software. Scale bars, 1 mm (left) or 50 μm (right). Arrows depict stained CD3^+^ cells. Determination of immunological hot tumors was set by the number of CD3^+^ cells/mm^2^. (D) Infiltration rate of tumors in peptide-treated mice was categorized in percentages of distance to the tumor center and number of CD3^+^ cells/mm^2^ in each category. Exponential regression analysis (One Phase Decay) was performed and the growth rate k plotted. Horizontal bars indicate the mean of each group with SEM. Significant differences between groups were determined using an one-way ANOVA with a Tukey’s multiple comparisons test (all Grouped Plots) a Mantel-Cox test (Kaplan-Meier plot), or a paired two-tailed t test (infiltration plot). Asterisks indicate significant difference with ∗*p* ≤ 0.05, ∗∗*p* ≤ 0.01, and ∗∗∗*p* ≤ 0.001.

Next we investigated whether the deconstruction of AAVLP capsids into CD4^+^ response-inducing peptides can improve the outcome when added *in trans* to the previously unsuccessful SIINFEKL peptide vaccination. We chose four potential MHC class II epitope peptides (i.e., p6, p7, p8, and p11) from the validation experiment (Figures 3D and 3E) and spiked them into SIINFEKL peptides at an equimolar ratio to the AAVLP capsid epitopes. Within a preventive tumor formation setting, we observed a significant improvement of success by SIINFEKL + AAVLP helper peptide vaccination compared with SIINFEKL alone (Figure S7A). Interestingly, the SIINFEKL + helper peptide vaccine performed similar to the AAVLP-SIINFEKL vaccine, reflected by longer survival of the animals. This effect seems to be CD8^+^ T cell mediated; we detected a significantly greater number of SIINFEKL-specific T cells in the spleens of surviving mice compared with non-survivors. Moreover, among them the rate of SIINFEKL T cells exhibiting a memory phenotype is higher in survivors vs. non-survivors, as well (Figure S7B). Prompted by these observations, we then scrutinized the effect of spiked helper peptides (SHPs) on vaccination in a therapeutic setting by application of the vaccine 4 days after melanoma cell injection. We observed reduced tumor growth after treating mice with AAVLP-SIINFEKL leading to significantly prolonged survival (Figure 4A), which was outperformed by the SHP vaccine (Figure 4B). In the SHP setting, 3 of 10 mice developed no tumors, while SIINFEKL treatment alone was ineffective. On the day of euthanization after tumor injection, we detected more SIINFEKL tetramer-positive T cells in the spleen and blood of the SHP vaccine group compared with the SIINFEKL-peptide vaccine alone group (Figure 4C). An unbiased software analysis of whole slide images of tumors from SIINFEKL- and SHP-vaccinated mice, obtained at the same time point, revealed a higher rate of infiltrating CD3^+^ T cells into the tumor tissue in the SHP group (Figure 4D). In addition, these T cells penetrated the tumor tissue deeper from the invasive margin to the tumor center (Figure 4E). To investigate the effect of helper peptide substitution on neo-antigen vaccination, we mixed SHP with the four top ranked MHC class I peptides to capsid-epitope equimolar amounts and vaccinated mice in a treatment setting (Figure 5A). We observed significantly delayed tumor growth and extended survival of mice vaccinated with neo-antigen peptides alone; the addition of SHP to the neo-antigen peptides yielded in a slightly better outcome, although the difference was not statistically significant (Figure 5B). As seen before, the SHP vaccine mix induced a higher T cell infiltration into tumors (Figure 5C) that also invaded deeper into the tumor tissue (Figure 5D). It is to note, that, although a cross-comparison of the treatment settings shows a trend to better outcome in animals treated with SIINFEKL-SHP over SIINFEKL-AAVLP, this is not statistically proven (Figure S8).

### Patient response to neo-antigen vaccination depends on MHC class II epitopes

**Figure 6:**
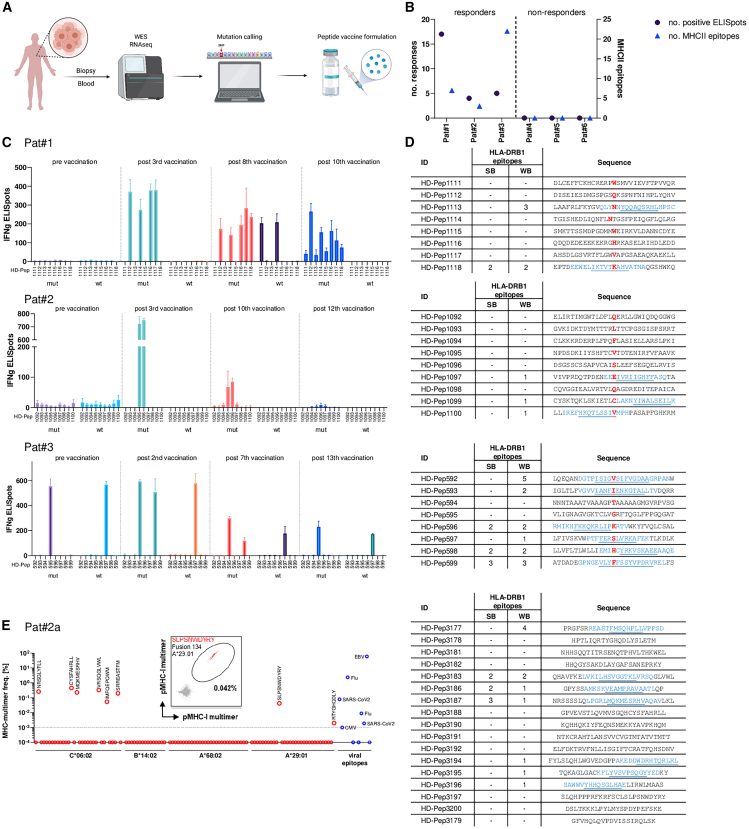
Patient response analysis after neo-antigen vaccination (A) Workflow of the patient treatment setting. Tumor biopsies and blood samples were acquired from six patients and WES as well as RNA sequencing of the tissue was performed. Mutation profiling combined with tumor expression data was adducted to choose up to nine neo-antigen synthetic long peptides (29mer) to be formulated in Montanide ISA51. Vaccination was performed intradermally in the preferred (left or right) upper arm of the patient. (B) Correlation plot of ELISpot responses after neo-antigen vaccination and number of called MHC class II epitopes within the peptide formulation. (C and D) Analysis of three patients responding to neo-antigen vaccination. (C) IFN-γ ELISpot counts of co-cultured APCs with autologous CD3^+^ T cells obtained before and after vaccination. Each ELISpot co-cultivation was performed in triplicates, mean number of IFN-γ counts ± SD is shown. (D) Tabular results of NetMHCIIpan 4.0 analysis of neo-antigen peptides used in vaccination. SB, strong binder; WB, weak binder. Mutations differing from WT peptide sequence are depicted in red, and predicted HLA-DRB1 epitopes are labeled in blue with the core underlined. (E) Analysis of CD8 T cell responses after *in vitro* stimulation (IVS) of PBMCs derived from patient 2 after vaccine adaptation. FACS analysis was performed using multimers that incorporate predicted MHC class I vaccine peptides.

To test the clinical relevance of MHC class II epitopes in a neo-antigen vaccine formulation, we retrospectively analyzed samples from six patients from our department (Table 1) who received a personalized tumor vaccine as a last line of therapy within an individualized treatment/compassionate use setting and after providing informed consent. Tumor biopsies and autologous peripheral blood mononuclear cells (PBMCs) underwent whole-exome sequencing (WES) and RNA sequencing. An established bioinformatics pipeline^31^ predicted single nucleotide variant (SNV) polymorphisms and epitope binding in the respective MHC-I proteins according to the patient’s individual HLA alleles. An individualized vaccine was formulated in Montanide ISA51 upon the physician’s choice and consisted of up to nine 29mer-long peptides per patient with the mutation centered (Figure 6A). Intradermal vaccinations of the patients were performed in the upper arm, initially bi-weekly with longer time span extension on later visits. Blood draws were carried out before and after vaccination on a regular basis for functional enzyme-linked immunospot assay (ELISpot) assays on the vaccinated peptides. A response was determined as positive when the IFN-γ counts for at least one mutated peptide exceeded the internal assay control and were undetectable in samples before vaccination (Figure 6C). We observed post-vaccination responses in three of the six patients; among them, the number of responses varied frequently (Figure 6B). We retrospectively analyzed the vaccine peptides for the occurrence of MHC class II (HLA-DRB1) epitopes of each patient and only found them in responding patients. The number of responses did not correlate with the number of detected epitopes, however. A detailed analysis of the individual patients (Figure 6C) revealed an initially high level of neo-antigen specific T cell responses that was detected up to the 10th round of vaccination but was declining in number of positive IFN-γ counts over time. The majority of responses was induced de novo with the exception of one peptide in patient 3 where reactive T cells were frequent pre-vaccination and a response detectable to the non-mutated control peptide. Interestingly, the neo-antigen-specific responses measured in patient 1 after the third and eighth vaccinations and for patient 2 throughout treatment affected peptide sequences that did not bear an MHC class II epitope. Thus, we may conclude that these responses were CD8^+^ mediated (Figure 6D). However, to support the hypothesis we performed a more detailed analysis on *in vitro* stimulated PBMCs derived from patient 2, who received an adapted vaccine due to declining measurable response and finally disease relapse. The novel vaccine was exclusively composed of neo-epitopes from predicted gene fusion peptides. By dual-color peptide MHC (pMHC) class I multimer FACS staining we detected six of eight CD8^+^ responses that were associated with the vaccine. Only one MHC class I epitope (MQKMESRHV, derived from HD-Pep3187) appeared to have a sequence overlap with a corresponding HLA-DRB1 epitope in the vaccine peptide. The other detected epitopes are not associated with a corresponding MHC class II epitope within the same vaccine sequence, e.g., SLPSNWDYRY, which is derived from HD-Pep3197 (Figure 6E). In summary, this clinical dataset supports the finding that sequence independent CD4^+^ help is needed to evoke neo-antigen-specific T cell responses.

**Table 1:** Patient characteristics

ID	Sex	Age	Location	Type	TNM (when vaccination was started)	Sites of metastasis (when vaccination was started)	Lines of systemic palliative therapies prior vaccine
Pat#1	female	27	breast	invasive ductal carcinoma	yT0 pN3 cM1	PUL, LYM	1
Pat#2	female	56	colorectal	adenocarcinoma	ypT3 pN2b cM1	PUL, OSS, BRA	2
Pat#3	male	51	tonsil	22 cell carcinoma	ypT2 pN0 cM1	PUL, BRA, HEP, OTH	1
Pat#4	female	37	breast	invasive ductal carcinoma, triple-negative	ypT2 ypN1a cM1	LYM	3
Pat#5	female	42	appendix	adenocarcinoma	pT4 pN0 pM1	PER, OTH	2
Pat#6	female	66	breast	invasive breast cancer	pT1c pN1a pM1	OSS, HEP, LYM	4

All patients had metastatic disease with no curative treatment options and received at least 1 line of systemic treatment before vaccination. TNM staging followed the guidelines of UICC, metastatic site abbreviations according to AJCC Cancer Staging Manual, 9th Edition.

## Discussion

In this study, we have tested and analyzed the feasibility and efficacy of an AAV-based VLP approach using neo-antigens in tumor prevention and treatment. We have shown that highly immunogenic epitopes can be integrated into the AAV capsid and induce a detectable CD8^+^ T cell response after one administration (Figure 1), which is comparable with existing methods.^9^ This effect is also transferable to a neo-antigen setting (Figure 2) that is currently tested in various phase 1 trials and represents the most individualized form of tumor treatment.^3^^,^^4^ We have analyzed in detail the superior vaccine effect of AAVLPs over MHC class I peptide vaccination alone and found that the AAV capsid provides essential MHC class II epitopes in its structure that may induce a licensing process of CD4 T cells toward CD8 T cells. Although this process is well known and has been described as a prerequisite for successful tumor vaccination,^32^ current studies so far only observed this effect on target, i.e., when MHC class II epitopes are encoded in the tumor antigen sequence itself. To our knowledge, we here describe for the first time that this licensing effect can be uncoupled from the tumor antigen sequence and, consequently, identified class II epitopes can be formulated as helper peptides that lead to a comparable outcome when used in preventive and therapeutic cancer vaccine settings (Figures 4 and 5). Recently, the importance of CD8 T cell licensing has also been reported for adoptive immunotherapy using chimeric antigen receptor T cells.^33^ In this paper, the authors state that the licensing occurs via triads that are formed between a CD8^+^, a CD4^+^, and a tumor cell that necessarily needs to present class I and class II epitopes at the same time and space. In our study, we can only speculate about the same spatial necessity when using the helper peptide approach, due to yet technical limitations in resolving single-cell peptidomics. An AAVLP tumor vaccine will be advantageous in that regard, as it will naturally deliver both epitope classes when taken up into a dendritic cell. Interestingly, when comparing the SIINFEKL + helper approach with the neo-antigen + helper vaccine, we observed a minor additional effect of helper peptides in the latter. Additionally, the outcome of this setting was more effective than the neo-antigen AAVLP vaccine alone. Most likely, this is due to the greater number of neo-antigen peptides, and therefore more potential class II epitopes, that could be formulated using peptides alone. The retrospective clinical data on a small cohort of individually treated patients that we present here (Figure 6) also support our findings obtained in the murine experiments. We found post-vaccine immune responses measured by ELISpot only in patients in whom the personalized peptide vaccine harbored MHC class II epitopes presented in the HLA-DRB1 allele. The importance of these CD4 T cell epitopes on the outcome of tumor vaccination has been described numerous times.^34^^,^^35^^,^^36^ Interestingly, we measured T cell responses by ELISpot and more profoundly also by MHC class I multimer FACS to peptides that do not harbor a class II epitope in their sequence, so we deduce that the inevitable T cell licensing must have occurred via another class II epitope present in the vaccine cocktail. In a very recent publication, Sultan et al.^37^ observed that a plethora of MHC class II epitopes in a tumor vaccine results in a reversed treatment effect. This is caused by CD4^+^ Tr1 cells that were entrained via class II epitopes, leading to the selective killing of conventional dendritic cells, thereby reducing the number of these APCs in the tumor tissue. Our finding of sequence independency to tumor T cell licensing using helper peptides may overcome that problem and may lead to a more efficient design of tumor vaccines.

## Materials and methods

### Cell culture

The human embryonic kidney (HEK) cell line 293T (ACC 635; DSMZ) was maintained at 37°C and 5% CO^2^ in a humidified atmosphere, cultured as a monolayer in DMEM supplemented with GlutaMax, 10% fetal calf serum, 50 U/mL penicillin, and 50 μg/mL streptavidin. The OVA-expressing murine melanoma cell line B16F10-OVA (kindly provided by Stefan Eichmüller) was maintained in complete RPMI.

### Plasmids

The adenoviral helper plasmid pDGΔVP [79], encoding adenoviral genes VA, E2A, and E4, was kindly provided by Oliver Müller. pMT-AAV2wtRC, encoding the WT sequences of AAV2 Rep and Cap proteins, was used to produce AAVLP2-WT. For the generation of AAVLP serotype 2 capsid mutants, antigen sequences were inserted into variants of pMT-187-XX2. pMT-187-XX2_(588)mut for insertion at aa588 contains MluI and AscI restriction sites spanning aa583–589 of the WT VP sequence. pMT-187-XX2_(453)mut for insertion at aa453 contains NheI and SpeI restriction sites spanning aa451–458. pMT-187-XX2_(453)mut_(588)mut contains both insertion sites. For integration of the antigen sequences, complementary single-stranded DNA oligonucleotides, with overhangs matching the restriction sites, were ordered from Sigma-Aldrich Chemie. Upon annealing of oligonucleotides, the sequence was inserted at the respective restriction site via ligation. Oligonucleotides for insertion at aa588 and aa453 are given in the oligo table.

AAVLP serotype 5 capsid mutants with insertion at aa578 were likewise generated by ligation of annealed DNA oligonucleotides. pMT-rep2cap5-SfiI578 encodes AAV serotype 2 rep and AAV serotype 5 cap with two SfiI restriction sites spanning aa576–579. Oligonucleotides 5′ TGGCGCGAGCATCATCAACTTCGAGAAGCTTGCCGccc 3′ and 5′ CGGCAAGCTTCTCGAAGTTGATGATGCTCGCGCCAgtg 3′ were used for insertion of SIINFEKL at aa578 of serotype 5 cap.

### AAVLP production, purification, and titration

AAVLPs were produced by transfection of HEK293T cells in a helper virus-free system, as described previously. Briefly, cells in 15-cm culture dishes were double transfected with the rep/cap plasmid pMT-187-XX2 and adenoviral helper plasmid pDGΔVP using the PEI transfection method. Three days after transfection, cells were harvested, washed in PBS, pelleted by centrifugation, and resuspended in AAVLP lysis buffer (50 mM Tris/HCl, 2 mM MgCL2, 150 mM NaCl, pH 8.5). After freeze-thaw lysis and benzonase treatment, AAVLPs were purified by iodixanol gradient. After purification, the sample was re-buffered and concentrated in AAVLP concentration buffer (100 mM sodium citrate, 10 mM Tris/HCl, 0.001% Pluronic F68) using Amicon Ultra-15 (50-kDa) filter units. Capsid titers of AAVLP preparations were determined by A20 ELISA (PROGEN) as described in the manufacturer’s instructions.

### Animals

Female C57BL/6 mice at 6–8 weeks of age were obtained from Janvier. Mice were allowed to acclimatize in the local animal facility (IBF, University of Heidelberg) for 1 week before the initiation of an experiment. All experiments were performed according to national guidelines and experimental protocols approved by the national authority (Regional Authority of Karlsruhe; official approval ID: 35–9185.81/G-84/18).

### Vaccination

Unless stated otherwise, 5.0E+11 AAVLPs of serotype 2 or equimolar amounts of peptides in 30 μL PBS were mixed 1:2 with Montanide ISA 51 (36362ZFL2R3; SEPPIC) and injected s.c. into the hock of mice under isoflurane anesthesia. Peptides used in this study were synthesized in house in the DKFZ Peptide Facility as described before. In some mice, AAVLPs were administered s.c. at the tail base or i.m. into the quadriceps femoris.

Some mice received AALVPs adjuvanted with 7.5 μg/mouse bis-(3′-5′)-cyclic dimeric adenosine monophosphate (c-di-AMP) (HY-12326; MedChemExpress) or 25 μg/mouse CpG ODN 2395 (CpG) (IAX-200-007-M001; Biomol), or a combination of either with Montanide ISA 51.

### Depletion of CD4^+^ T cells

CD4^+^ T cells were depleted in mice by intraperitoneal injection of anti-CD4 (BE0003-1; BioXCell), while control mice received rat immunoglobulin G2b isotype control (BE0090; BioXCell). Antibodies were injected with a dose of 250 μg per mouse 2 days before vaccination and 100 μg per mouse 1 and 4 days after vaccination.

### *In vivo* tumor growth

Cultured B16F10-OVA tumor cells were detached in trypsin, washed twice with PBS and injected s.c. into the flank with 2.0E+05 cells per mice. The length and width of the developing tumors were determined in blinded measurements three times a week using a caliper. The tumor volume was calculated for each mouse as: volume = length × (width)^2^ × 0.5236. Mice were euthanized when tumors exceeded 12 mm on any side, necrotic wounds emerged or mice showed clear signs of distress.

### Intracellular staining of peptide-stimulated splenocytes

Unless stated otherwise, spleens were isolated from mice 3 weeks after vaccination. Splenocytes were stimulated with 2 μg/mL peptide in complete RPMI containing 50 μM 2-mercaptoethanol under normal cell culture conditions. Peptides included chicken Ova257-264 (SIINFEKL; S7951; Sigma-Aldrich), LCMV_NP396-404 (FQPQNGQFI; DKFZ core facility) and FLAG peptide (DYKDDDDK; DKFZ core facility). After 1 h incubation, 5 μg/mL brefeldin and 2 μM monensin were added and incubated for additional 5 h. For flow cytometry, dead cells were labeled using Zombie Aqua Fixable Viability Kit (423102; BioLegend) and cells were stained with fluorescent-tagged surface antibodies PerCP/Cy5.5 anti-mouse CD8 (1:100) (100734; BioLegend), FITC anti-mouse CD4 (1:200) (100406; BioLegend), and PacificBlue anti-mouse B220 (1:100) (103227; BioLegend). Upon fixation and permeabilization using the Cytofix/Cytoperm Fixation/Permeabilization Kit (554714; BD), intracellular cytokines were stained intracellularly with APC anti-mouse IFN-γ (1:50) (505810, BioLegend) and PE/Cy7 anti-mouse TNF-α (1:80) (506324; BioLegend). Fluorescence was detected by flow cytometry using a BD FACSCanto II.

### Tetramer staining

Tetramer staining was performed on immune cells derived from blood, spleen, axillary lymph nodes or inguinal lymph nodes. Blood samples were collected in EDTA-containing tubes and erythrocytes were lysed in red blood cell lysis buffer. Cells from spleen and lymph nodes were transformed into single cell suspension by passaging through a 100-μm cell strainer. Dead cells were labeled using Zombie Aqua Fixable Viability Kit (423102; BioLegend) and SIINFEKL-specific CD8^+^ T cells were labeled with APC-coupled H-2Kb/SIINFEKL MHC tetramer (MKb-001; SIINFEKL) in the presence of 50 nM dasatinib. After staining for surface markers using FITC anti-mouse CD8 (1:20) (D271.4; MBL), PE anti-mouse CD3 (1:100) (100307; BioLegend), and PacificBlue anti-mouse B220 (1:100) (103227; BioLegend), cells were analyzed by flow cytometry using a BD FACSCanto II.

### *In silico* prediction of AAVLP-derived MHC class II epitopes

Potential MHC class II eptitopes in the AAVLP capsid were determined using NetMHCII2.3 and NetMHCIIpan 4.0. The capsid sequence of AAVLP-SIINFEKL (SIINFEKL inserted at aa588) was analyzed for the binding of 15-mers to H-2-IAb. All predicted strong binders (2% rank) were designed as peptides, whereas overlapping 15-mers with the same core sequence were merged, resulting in 11 peptides (p1–p11) of 16–27 aa. One peptide predicted to be a non-binder was included as a negative control (p0). All peptides were produced in-house at the DKFZ Peptide Facility.

### Patients

Selected patients with advanced disease were treated at the Department of Medical Oncology at the NCT in Heidelberg (Table 1) and gave written informed consent for the collection and investigational use of blood and tissue samples. This procedure was approved by the Internal Review Board of the University Hospital Heidelberg (approval no. S-207/2005). All activities were conducted in compliance with the Declaration of Helsinki.

DNA and RNA from tumor tissue and corresponding blood-derived non-tumor DNA were profiled using WES and whole transcriptome sequencing as previously described.^31^ Briefly, somatic non-synonymous SNVs were identified based on DNA sequencing data (WES) and the quantification of gene expression based on RNA sequencing data were performed with the DKFZ Omics Data Core Facility supported analyses module “One touch platform (OTP).” Neoepitopes were predicted with our in-house pipeline utilizing netMHCpan4.1 and netMHCstabpan1.0 for MHC class I epitope prediction, according to patient-specific HLA type, which are predicted by OptiType and Kourami. Prediction results are ranked by binding affinity, binding stability, and mRNA expression levels. Long peptides spanning upstream and downstream aa from the mutation site were synthesized as 25–29 mers by Fmoc chemistry using a fully automated multiple synthesizer (Syro II, MultiSynTech). Synthesis was carried out on preloaded Wang-resins with 2-(1H-Benzotriazole-1-yl)-1,1,3,3-tetramethyluronium hexafluorophosphate as a coupling agent. Montanide ISA51 VG (SEPPIC) was used as adjuvant to form a water-in-oil emulsion, mixed with the peptide solution containing 60 μg per peptide each (aqueous phase, 10% DMSO) in a 50:50 ratio. In individual treatment approaches, the peptide mixes were administered intradermally. Blood samples were collected before vaccination and longitudinally.

### INF-γ screening by ELISpot assays

To obtain APCs, PBMCs were isolated by Ficoll gradient centrifugation, monocytes were depleted by adherent plating and further cultivated in X-Vivo 20 medium containing IL-4 (60 IU/mL) and GM-CSF (560 IU/mL). The remaining lymphocytes were cultivated in X-Vivo 20 medium with IL-2 (100 IU/mL) and IL-4 (60 IU/mL). To test T cell reactivity, monocytes were pulsed ON with 10 μg/mL long peptides or short peptides and co-cultured the next day with autologous lymphocytes in filter plates with a 0.45-μm pore size Mixed Cellulose Esters membrane (Millipore), plating 100,000 monocytes and 20,000 T cells per well in triplicate or duplicate, in screening or validation assays, respectively. Anti-CD3 coated wells (10 μg/mL, clone OKT3, Biolegend) were used as positive controls. Viral peptide pools (PepTivator CEF MHC Class I Plus, Miltenyi Biotec) as internal controls and negative controls (unstimulated cells and wells with only media) were included in every assay. INF-γ detection was performed using Human IFN-γ ELISpot basic kit (ALP) (Mabtech) following the manufacturer’s protocol. Images were acquired and analyzed with ImmunoSpot Analyzer (C.T.L. Europe).

### *In vitro* stimulation and multimer staining of PBMCs

*In vitro* stimulation was performed as previously described,^38^ with slight modifications. CD40L-B cells were pulsed ON with long peptides and co-cultured the next day with enriched CD3^+^ (Pan T cell isolation kit, Miltenyi Biotec) or CD8^+^ (REAlease CD8 MicroBead Kit, Milenyi Biotec) in T cell medium containing 30 ng/mL interleukin (IL)-21. Cells were incubated at 37°C for 72 h, after which time the cultures were fed with fresh T cell medium containing 30 ng/mL IL-21, 3000 IU/mL IL-2, 2.5 ng/mL IL-15, and 2.5 ng/mL IL-7. The splits were performed after 72 h. After the second split, T cell reactivity was measured.

Multimer libraries of pMHC-I complexes loaded with individual short peptides were generated as described before,^39^ and a staining procedure was undertaken. Briefly, cultured *ex vivo* CD8^+^ T cells were washed once with DPBS/100 nM dasatinib and were then labeled with the Zombie Aqua Fixable Viability Kit (BioLegend 423102) 1:300 for 10 min at room temperature (RT) in DPBS +100 nM dasatinib. Next, one volume of pMHC staining buffer + Human TruStain FcX (Fc receptor blocking solution, BioLegend 422302) was added 1:50 (v/v) after incubation for 5 min at RT. Cells were then stained with prepared pMHC-I multimer libraries at RT for 25 min. After one wash, cells were stained using a cocktail containing optimally titrated antibodies (all from BioLegend) against human CD14 (M5E2, Cat. No. 301842), CD16 (3G8, 302048), CD19 (H1B19, 302242), and CD335 (9E2, 331924) (all Brilliant Violet 510-conjugated, defined as dump channel); CD8 (SK1, BioLegend 344714) APC-Cy7, and CD3 (UCHT-1) Alexa Fluor 700 (BioLegend 300424).

### Immunohistochemistry

Tissue sections were prepared from formalin-fixed, paraffin-embedded tissue (3 μm). Sections were stained with the anti-CD3 mAb SP7 and BOND Polymer Refine Red Detection kit. The complete staining procedure was carried out on a BOND-RX (Leica) following the manufacturer’s protocol.

### Software

Whole slide images were acquired using a VS200 slide scanner (Olympus) in two consecutive runs followed by co-registration of all images. The density and distribution of immune cells in complete microscopic images of full tissue sections were semi-automatically analyzed using HALO software (Indica Lab). Scientific graphs were created using GraphPad Prism 8.0.2, all non-scientific sketches were drawn using BioRender under CC-BY 4.0 license, the AAV visualization was done using UCSF ChimeraX based on PDB entry no. 1LP3.

### Statistical analyses

Statistical analysis were performed using GraphPad Prism 8.0.2. Significant differences were determined using a two-tailed t test, one-way ANOVA, or two-way ANOVA with a Dunnett’s multiple comparison test. Unless indicated otherwise, asterisks indicate significant differences to the negative control with ∗*p* ≤ 0.05, ∗∗*p* ≤ 0.01, and ∗∗∗*p* ≤ 0.001.

## Data availability

The datasets generated and analyzed during this study are available from the corresponding author upon reasonable request and with respect to clinical data following the regulations of European Data Protection laws.

## Acknowledgments

We thank Zhiqin Huang for his contribution to the development of our in-house neo-epitope prediction pipeline. We are thankful to Claudia Luckner-Minden, Iris Kaiser, Annette Köster, and Nina Wilhelm for the excellent technical assistance.

## Author contributions

L.N., S.U-S., P.S., B.L., M.M., and I.Z. designed experiments; L.N., K.v.W., A.T., B.L., and M.M. performed experiments; L.N., S.U-S., B.L., and P.S. analyzed data; S.E. manufactured peptides; J.S. and D.J. treated patients; I.Z. and D.J. supervised the work and provided resources; P.S. wrote the manuscript, L.N., S.U-S., J.K., I.Z., and D.J. revised the manuscript. L.N. and S.U.S. contributed equally to this work.

## Declaration of interests

All authors declare no competing interests.

## Supplemental information

Document S1. Figures S1–S8

Document S2. Article plus supplemental information
