# Supplementary material for: Neo-antigen tumor vaccination depends on CD4-licensing conveyed by adeno-associated virus like particles
Source: Mol Ther. 2025 Jul 16;33(10):5003–16. doi: 10.1016/j.ymthe.2025.07.014 (PMC12848249; doi:10.1016/j.ymthe.2025.07.014)
Supplement: Document S1. Figures S1–S8 [file mmc1.pdf]

YMTHE, Volume *33*

Supplemental Information

Neo-antigen tumor vaccination depends on CD4-licensing conveyed by adeno-associated virus like particles

Lasse Neukirch, Silke Uhrig-Schmidt, Katharina von Werthern, Alexandra Tuch, Joscha A. Kraske, Yanhong Lyu, Benedicte Lenoir, Stefan B. Eichmüller, Marten Meyer, Inka Zörnig, Dirk Jäger, and Patrick Schmidt

Figure S1

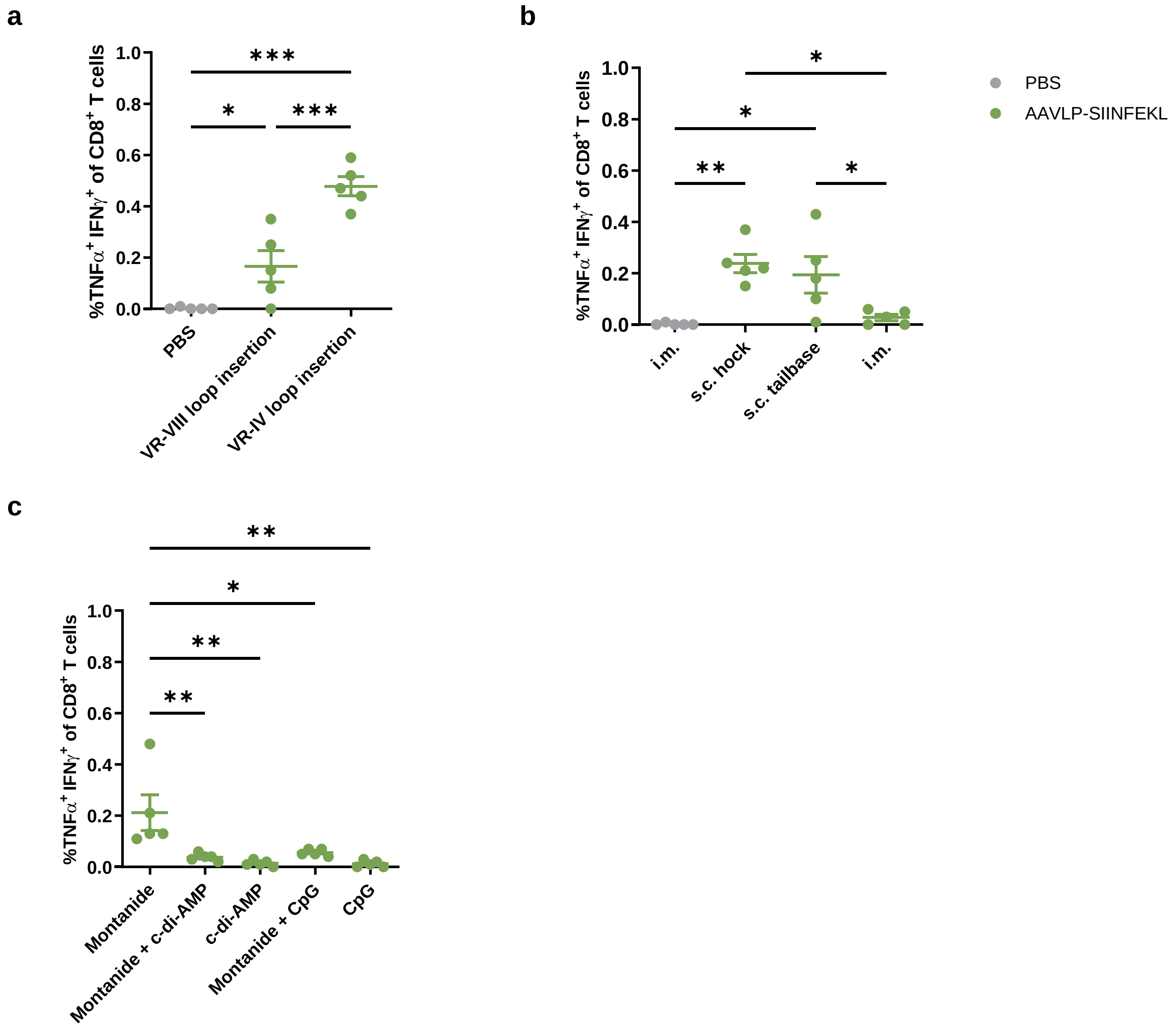

Figure S1: Optimization of AAVLP vaccine by comparing injection routes, adjuvants, and antigen insertion sites. All mice were injected with 5.0E+11 SIINFEKL-displaying AAVLPs. Antigen-specific CD8+ T cell responses were determined after three weeks by stimulating splenocytes with SIINFEKL peptide, followed by intracellular staining of activation markers TNFα and IFNγ. **a** Antigen insertion site: Mice were vaccinated with AAVLP-SIINFEKL, in which the SIINFEKL antigen was inserted in the VR-VIII loop around aa588 or in the VR-IV loop around amino acid 453 of the capsid protein VP1. PBS injected mice served as negative controls. **b** Injection route: AAVLP-SIINFEKL was injected s.c. into the hock, s.c. at the tailbase or i.m.. Mice injected i.m. with PBS served as negative controls. **c** Adjuvants: AAVLP-SIINFEKL administered s.c. into the hock was adjuvanted with Montanide ISA 51, c-di-AMP, CpG ODN 2395 or combinations thereof. Mice injected with AAVLP-SIINFEKL without adjuvant served as controls. Horizontal bars indicate the mean of each group (n=5) with SEM. Significant differences between groups were determined using a One-way ANOVA with a Tukey's multiple comparisons test. Asterisks indicate significant difference with * ($P \leq 0.05$); ** ($P \leq 0.01$); *** ($P \leq 0.001$).

Figure S2

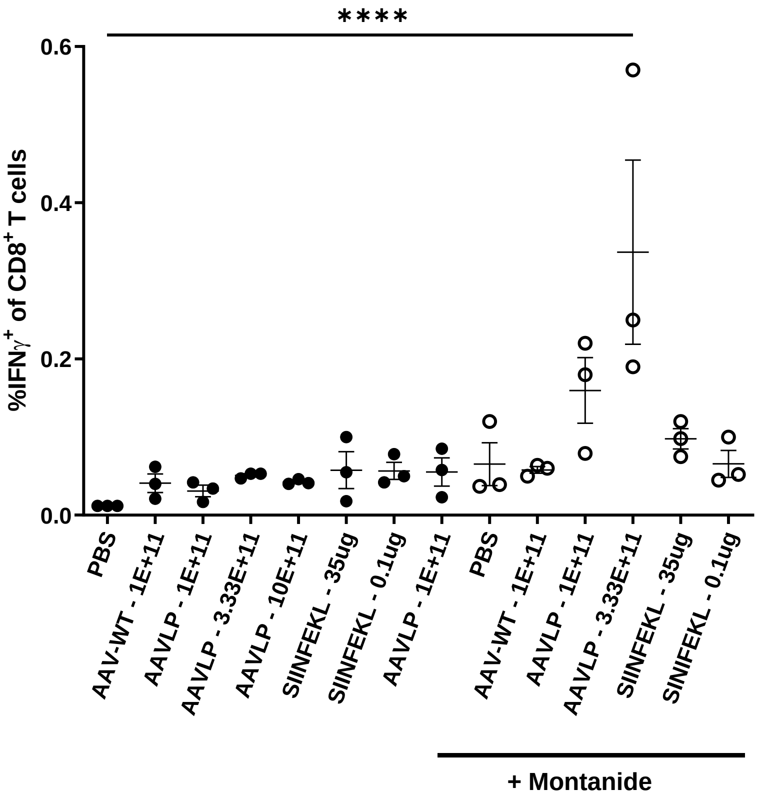

Figure S2: Dose optimization of AAVLPs. Mice were injected s.c. hock with varying amounts of SIINFEKL-displaying AAVLPs. Equimolar amounts of SIINFEKL peptide (35µg correspond to 3.33E+11 VLPs and 0.1µg correspond to 1E+11 VLPs) served as comparator, injection was performed with or without formulation in Montanide ISA51. Antigen-specific CD8+ T cell responses were determined after three weeks by stimulating splenocytes with SIINFEKL peptide, followed by intracellular staining of the activation marker IFNγ. Horizontal bars indicate the mean of each group (n=3) with SEM. Significant differences between groups were determined using a One-way ANOVA with a Tukey's multiple comparisons test. Asterisks indicate significant difference with **** (P ≤ 0.0001).

Figure S3

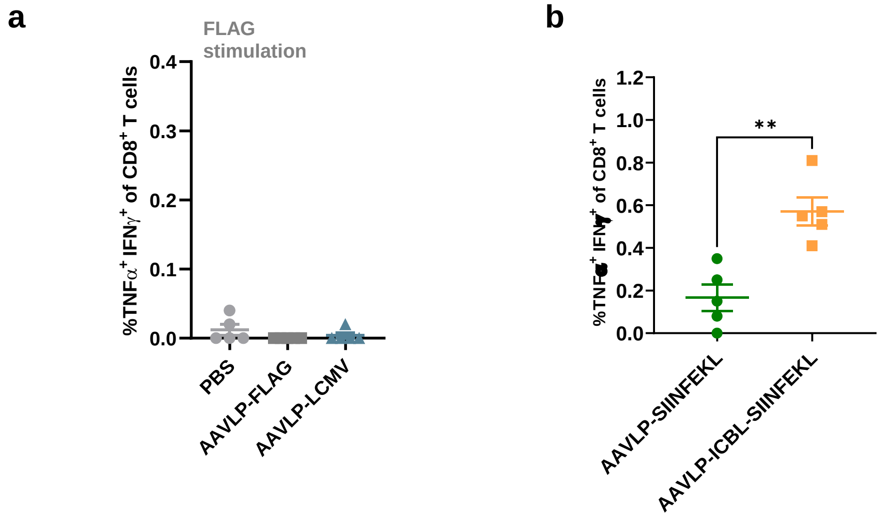

Figure S3: Versatility of AAVLP vaccination strategy. a Mice were vaccinated with 5.0E+11 capsids of AAVLP-LCMV s.c. into the hock in Montanide ISA 51. AAVLP-LCMV displayed the CD8+ T cell epitope NP396-404 of LCMV in the VR-VIII loop. Mice injected with PBS or AAVLP with inserted FLAG tag served as negative controls. Antigen-specific CD8+ T cell responses were determined after three weeks by stimulating splenocytes with FLAG (negative control) or LCMV NP396-404 peptide, followed by intracellular staining of activation markers TNFα and IFNγ. Horizontal bars indicate the mean of each group (n=5) with SEM. Significant differences between groups were determined using a One-way ANOVA with a Tukey's multiple comparisons test. Asterisks indicate significant difference with *** ($P \leq 0.001$). **b** Mice were vaccinated with 5.0E+11 AAVLP-SIINFEKL (SIINFEKL in VR-VIII loop) or AAVLP-ICBL-SIINFEKL, in which the J-ICBL peptide (DLLKNGERIEKVE) was inserted in the VR-IV loop and SIINFKEL in the VR-VIII loop. Antigen-specific CD8+ T cell responses were determined after three weeks by stimulating splenocytes with SIINFEKL peptide, followed by intracellular staining of activation markers TNFα and IFNγ. Horizontal bars indicate the mean of each group (n=5) with SEM. Significant differences between groups were determined using a two-tailed t-test. Asterisks indicate significant difference with ** ($P \leq 0.01$)

Figure S4

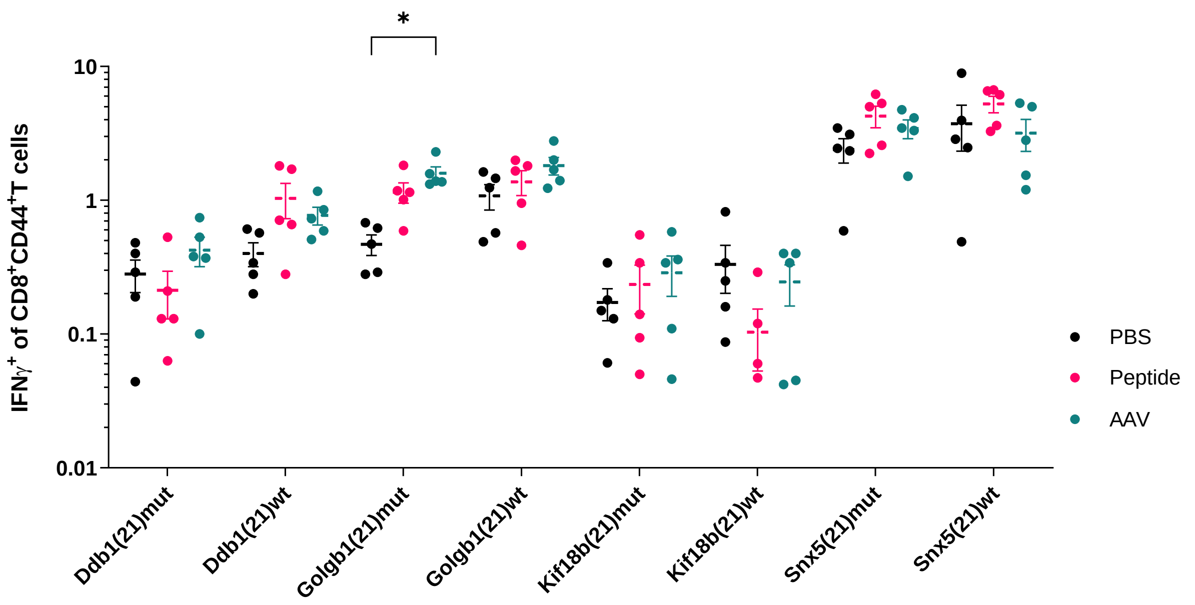

Figure S4: Response to neo-antigen vaccination. Mice were injected s.c. hock with varying amounts of neo-antigen-displaying AAVLPs. Equimolar amounts of neo-antigen peptide pools (35µg correspond to 3.33E+11 VLPs and 0.1µg correspond to 1E+11 VLPs) served as comparator, injection was performed with formulation in Montanide ISA51. Antigen-specific CD8+ T cell responses were determined after three weeks by stimulating splenocytes with each of the neo-antigen peptide of the pool (mut) and its respective non-mutated version (wt), followed by intracellular staining of the activation marker IFNγ. Horizontal bars indicate the mean of each group (n=3) with SEM. Significant differences between groups were determined using a One-way ANOVA with a Tukey's multiple comparisons test. Asterisks indicate significant difference with **** ($P \leq 0.0001$).

Figure S5

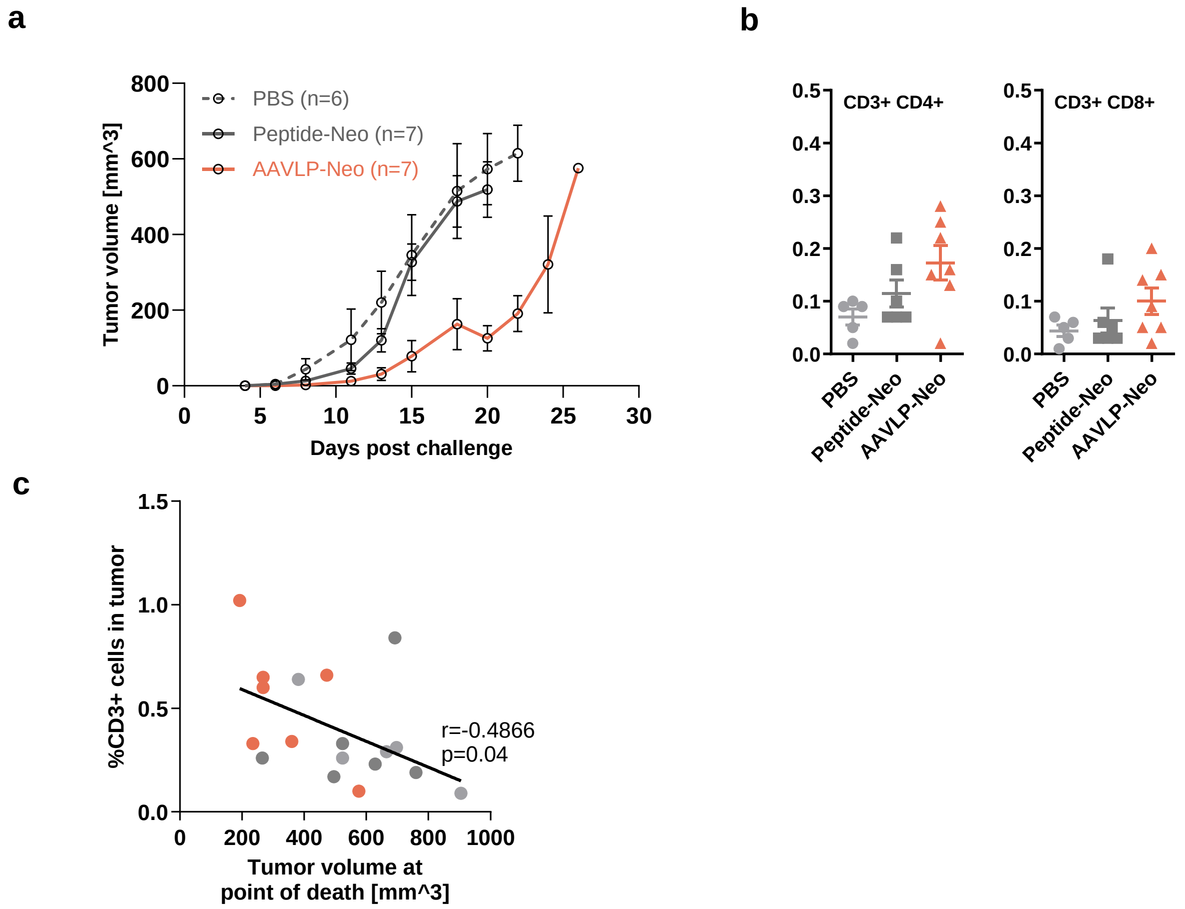

Figure S5: Reduced tumor growth after neo-antigen-AAVLP vaccination. a Mean tumor growth over time for the vaccination experiment of Fig.2, error bars represent SEM. **b** Detailed analysis of FACS plots derived from Fig. 2b. **c** Tumors from euthanized mice were explanted and single cell suspension of tumor cells were analyzed by FACS for frequency of CD3+ T cells. Graph plots the correlation between tumor size at point of euthanization and the frequency of CD3+ cells in tumor suspension. Color coding equals to analysis in Fig.2. Linear regression analysis was performed and the Pearson r value is shown.

Figure S6

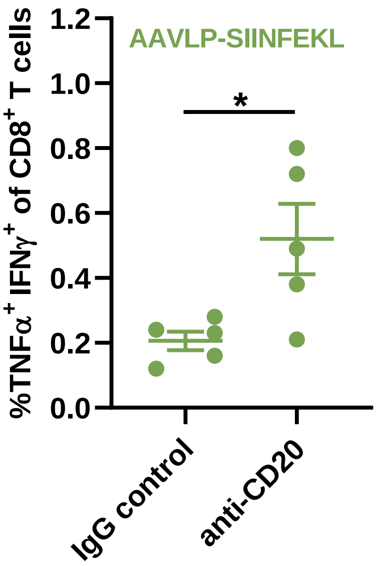

Figure S6: B cell depletion. By injecting anti-CD20, B cells were depleted during s.c. hock vaccination with 5.0E+11 capsids of AAVLP-SIINFEKL in Montanide ISA 51. AAVLP-SIINFEKL vaccinated mice injected with an IgG isotype control served as controls. Antigen-specific CD8+ T cell responses were determined after three weeks by stimulating splenocytes with SIINFEKL peptide, followed by intracellular staining of activation markers TNFα and IFNγ. Horizontal bars indicate the mean of each group (n=5) with SEM. Significant differences determined by Two-tailed t-test. Asterisks indicate significant difference with * ($P \leq 0.05$).

Figure S7

a

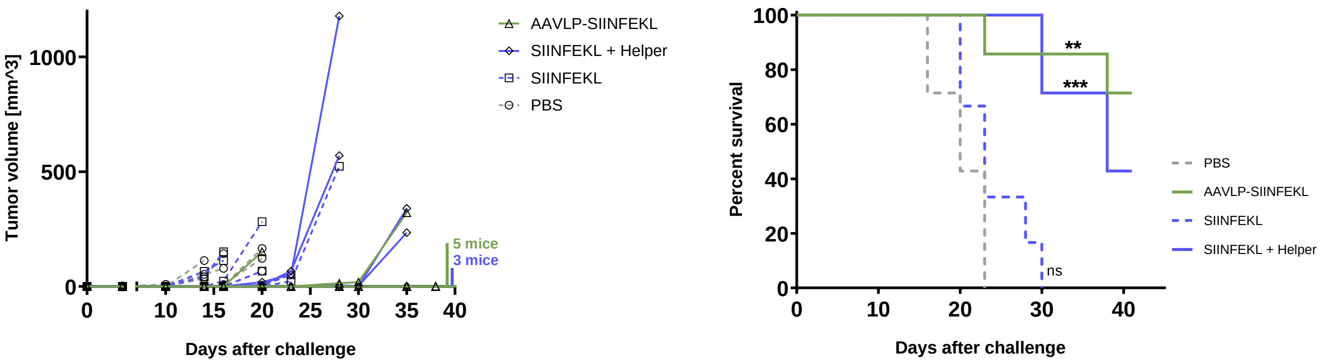

b

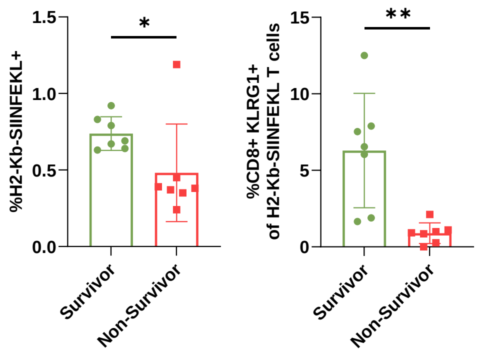

Figure S7: Effect of peptide vaccination spiked with AAV helper peptides in a preventive setting. a Mice were vaccinated with 5.0E+11 AAVLP-SIINFEKL or equimolar amounts of SIINFEKL peptides or equimolar amounts of mixed helper peptides (p6,7,8,11) together with SIINFEKL. Three weeks after vaccination all mice received one s.c. injection of 2.0E+5 B16F10 melanoma cells into the left flank. Tumor growth and survival was monitored over 40 days and plotted. **b** Spleens of euthanized or surviving mice at day40 were explanted minced and splenocytes analyzed by tetramer FACS for the occurrence of SIINFEKL-TCR positive cells (left panel). These cells were then subgated and stained for the effector T cell marker KLRG1 (right panel).

Figure S8

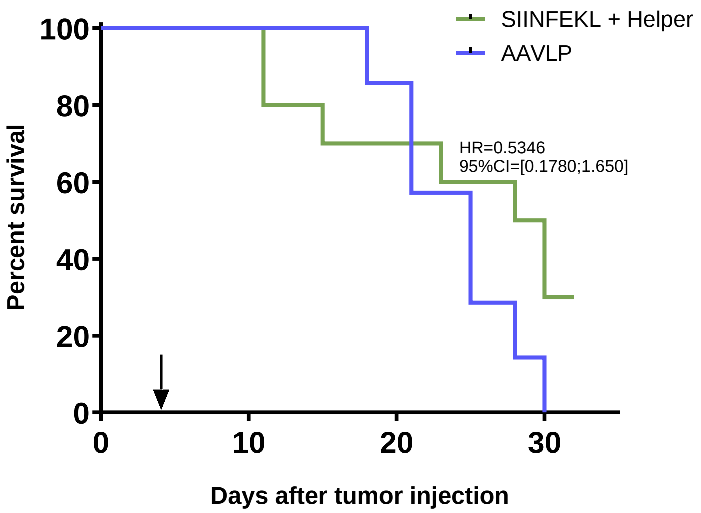

Figure S8: Cross comparison of peptide vaccination spiked with AAV helper peptides and AAVLPs in a treatment setting. All mice received one s.c. injection of 2.0E+5 B16F10 melanoma cells into the left flank at day 0. Mice were treated at day 4 with 5.0E+11 AAVLP-SIINFEKL or equimolar amounts of SIINFEKL peptides or equimolar amounts of mixed helper peptides (p6,7,8,11) together with SIINFEKL. Survival was monitored over 35 days and plotted. The Log-Rank Mantel-Cox test (Kaplan-Meier-Plot) was applied and Hazard Ratio within the 95% Confidence Interval was calculated.
